# Case report: Thoracolumbar spinal stenosis associated with alkaptonuria

**DOI:** 10.3389/fsurg.2022.1040715

**Published:** 2023-01-06

**Authors:** Hong Ding, Liang Wang, Gan-Jun Feng, Yue-Ming Song, Li-Min Liu

**Affiliations:** Department of Orthopedics, Orthopedic Research Institute, West China Hospital, Sichuan University, Chengdu, China

**Keywords:** alkaptonuria, thoracolumbar spinal stenosis, case report, surgery, kyphosis

## Abstract

**Background:**

Alkaptonuria is a rare autosomal genetic disorder with an incidence of about 1 in 1 million per year. Spinal involvement often manifests in the later stages of the disease. However, this is the first report of the presentation of thoracolumbar spinal stenosis.

**Case presentation:**

We report the case of a 61-year-old female patient with significant thoracolumbar stenosis symptoms. The patient had obvious kyphosis with preoperative lower extremity muscle strength grade 2/5. Symptoms and imaging signs initially suggested ankylosing spondylitis. This patient was classified into motor incomplete injury (ASIA C). However, the patient was found to have melanin deposits on the sclera and skin, and the urine was darkened at rest. CT and MRI both suggested no bone bridge connection between vertebrae, which was the key difference between ankylosing spondylitis and alkaptonuria in imaging. Most importantly, urine specimen testing and intraoperative pathology demonstrated alkaptonuria. The patient underwent spinal decompression and vertebral body fixation. Postoperative recovery was good: the patient had significantly relieved pain and could stand and walk.

**Conclusion:**

This case is the first report of thoracolumbar spinal stenosis associated with alkaptonuria involving the spine.

## Introduction

Alkaptonuria (AKU) is a rare genetic disease with an incidence of about 1 in 1 million per year (1). The early manifestation of AKU involves the darkening of urine after resting. The progression appears as melanosis of the skin and sclera. In the late stages of disease progression, homogeneous acid (HGA) deposits in tissues such as cartilage, tendons, and ligaments lead to the degeneration of the spine and large peripheral joints. Spinal canal stenosis frequently occurs in the cervical and lumbar spine but rarely in the thoracolumbar segment (2).

We describe here for the first time the case of a 61-year-old patient with AKU presenting with thoracolumbar spinal stenosis.

## Case report

A 61-year-old female patient complained of recurrent low back pain for more than 10 years and pain and numbness in both legs for approximately 8 months. We found that the patient's parents are married cousins, suggesting that the patient may have a genetic disorder. Physical examination revealed that the patient had significant kyphosis. Melanosis could be seen in both sclerae and both auricles. There were hypoesthesia and hypoalgesia below the T10 dermatome. The muscle strength for hip flexion, knee extension, and knee flexion was grade 3/5, while ankle dorsal extension and metatarsal flexion were grade 2/5. The patient exhibited hyperreflexia of both Achilles tendons and knees; furthermore, Babinski's sign, Gordon's sign, Oppenheim's sign, and the four-figure test were positive on both sides. This patient was classified into motor incomplete injury (ASIA C). On imaging examination, plain lumbar spine radiographs demonstrated that all the lumbar disc spaces were narrow with signs of osteoporosis. Magnetic resonance imaging (MRI) revealed spinal stenoses of the T10/11, L1/2, and L2/3 segments. Laboratory examination depicted that the fresh urine was light yellow and gradually turned dark brown after a period of time. Pathological examination revealed melanin deposits in the intervertebral disc and ligament tissues.

The patient underwent adequate intraoperative spinal canal decompression and was fixed with pedicle screws. At operation, we found that the bone junctions of the supraspinous ligament, interspinous ligament, and ligamentum flavum were blackened. Severe T10/11 disc degeneration was noted, with no significant distinction between the nucleus pulposus and the adjacent tissue.

The patient experienced remarkable relief and was discharged 1 week after the operation. The patient's muscle strength returned to grade 4/5 after surgery, and she could stand and walk independently. A review is scheduled for 3 months after surgery and lumbosacral surgery at an optional date, depending on the patient's condition.

## Discussion

This was the first description of alkaptonuria presenting as thoracic spinal stenosis. AKU is an autosomal recessive hereditary disease with an incidence of about 1 in 1 million per year (1, 3). This patient presented with symptoms similar to those of ankylosing spondylitis (AS) and was eventually diagnosed as an extremely rare case of alkaptonuria, which resulted in good recovery through surgery, so we feel it is important to report this case. The early clinical manifestations of AKU are mainly dark urine or deepening of urine color and hyperpigmentation, mostly in the sclera and external auricle. As the disease progresses, the deposits lead to stiffness of the connecting tissues, premature degeneration of the spinal joints, and labral osteophytes at the edges of the vertebral column (4). The characteristic imaging features include extensive intervertebral space narrowing and pancake-like disc calcification, sometimes with disc vacuum (5, 6).

**Figure 1:**
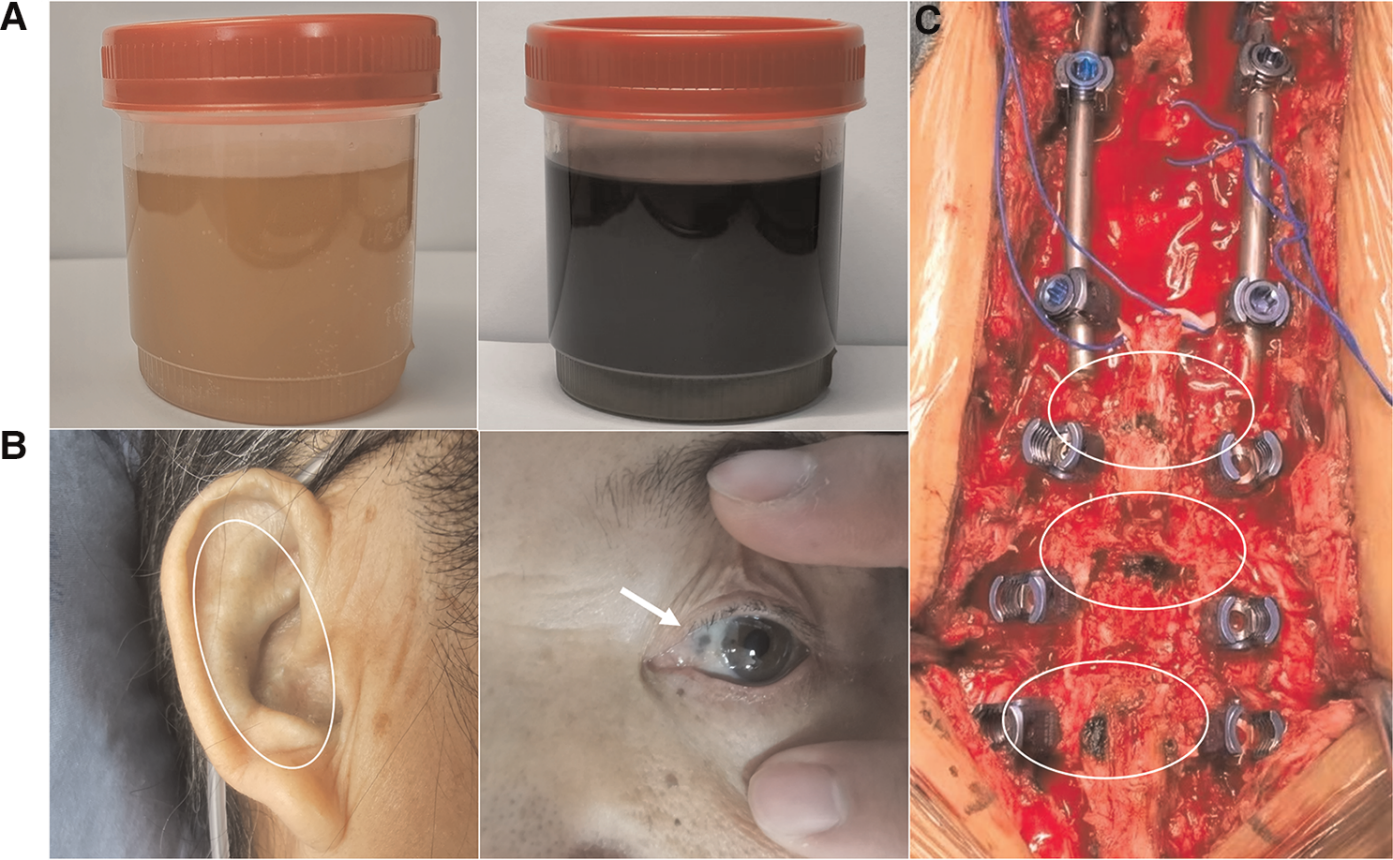
Patient's urine darkened after resting (**A**); melanin deposition in the sclera and external auricle (**B**); intraoperatively, the bony junction of the interspinous ligament, supraspinous ligament, and ligamentum flavum were black (**C**).

**Figure 2:**
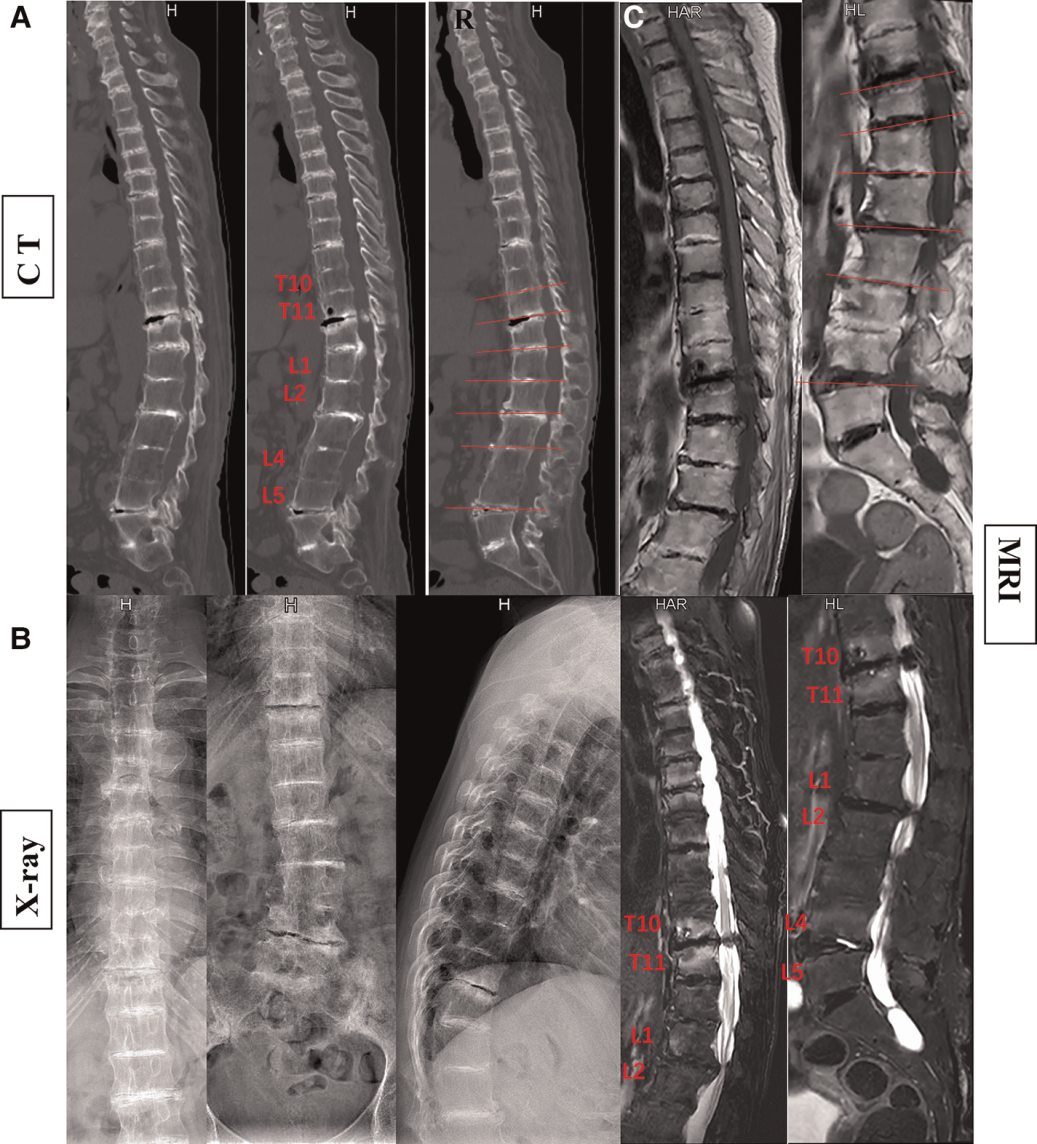
The patient's preoperative imaging examination revealed kyphosis and spinal stenosis. Disruption and stenosis of the intervertebral disc with labral changes. Intervertebral ossification dysplasia, vertebral body without ossification connected (**A**, CT; **B**, x-ray; **C**, MRI).

In the currently reported patient, melanin deposition in the sclera and external auricle and darkening of the urine after resting were observed. In the operation, the T10/11 disc was found to be severely degenerated, and the nucleus pulposus was indistinguishable from the adjacent tissue. Intraoperatively, parts of the interspinous ligament, supraspinous ligament, and ligamentum flavum were found to be darkened (Figure 1). Disc herniation has been previously reported in patients with AKU (7, 8), but severe thoracolumbar spinal canal stenosis is rare (9). Alkaptonuria patients are easily misdiagnosed as AS. AS can be followed by disc fibrosis and calcification, bony ankylosis of the spine, and the characteristic bamboo spine (10). Increased brittleness of spinal bone due to bony ankylosis of the spine and vertebral osteoporosis in late AS, combined with a stress increase in the thoracolumbar segment, leads to a fracture called an Andersson lesion (11). However, imaging data revealed that the patient had multiple intervertebral degenerations, including decreased intervertebral height, destruction of the upper and lower endplates, and low signal in the nucleus pulposus region on T2 images owing to atrophy and dehydration of the nucleus pulposus. No significant vertebral fusion was found in the spine (Figure 2). This is the point of differentiation from the presentation of patients with AS.

**Figure 3:**
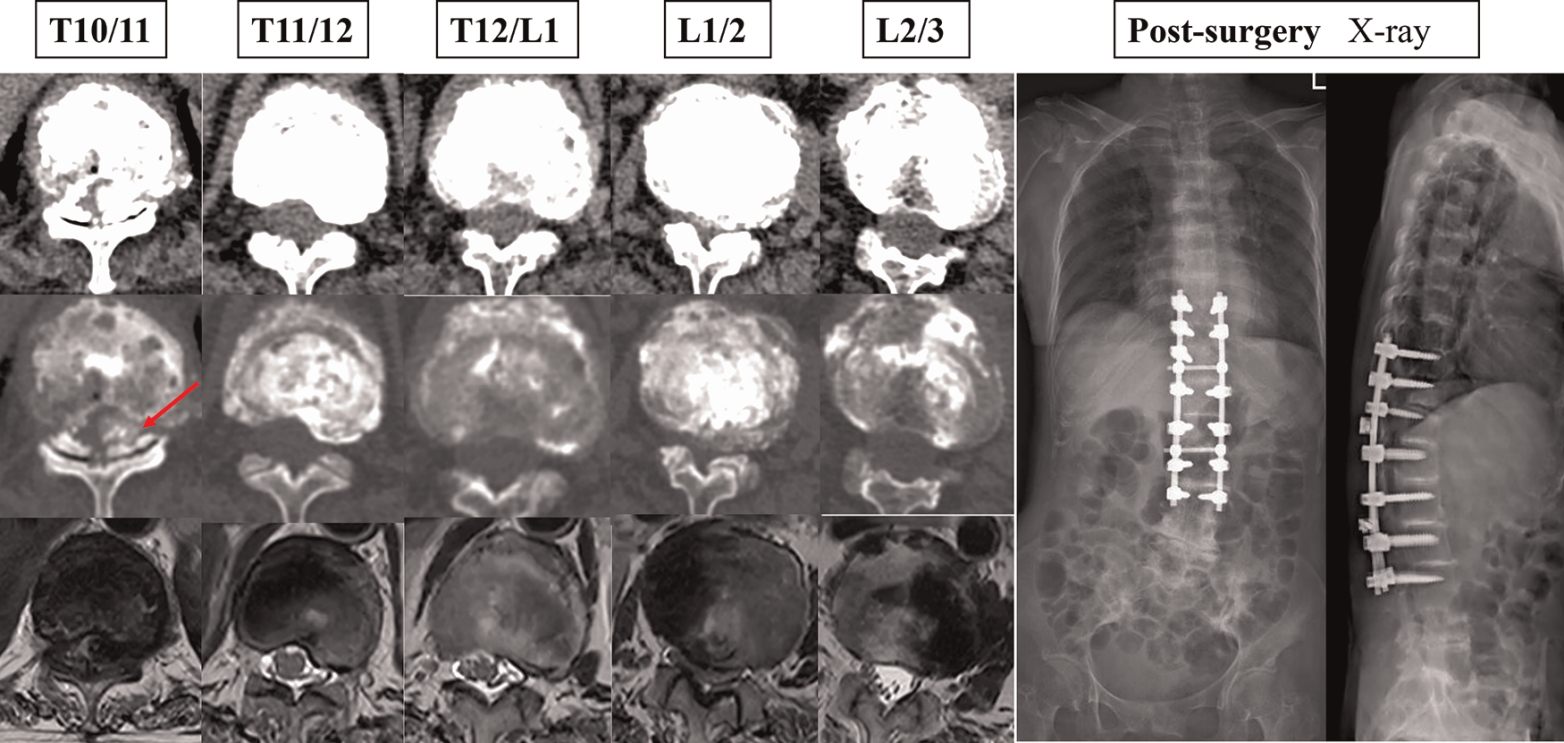
The patient's preoperative cross-sectional imaging examination and postoperative x-ray maps.

Narrowing of the spinal canal or foramina is a common finding in spine imaging of the elderly. Only when symptoms of neurogenic claudication and/or cervical myelopathy are present is a spinal stenosis diagnosis made, of the lumbar spine, cervical spine, or both (only very rarely is the thoracic spine involved) (2). The patient in the case report had significant preoperative spinal cord symptoms, was unable to walk independently, was pushed in a wheelchair, and had preoperative muscle strength of grade 2/5. Postoperatively, she could stand and walk, and her muscle strength returned to grade 4/5. Thoracic spinal stenosis is a rare entity for which the incidence is unknown (12). Spinal stenosis can be classified etiologically into two categories: congenital and acquired (13). Most patients will have acquired canal stenosis, often due to degenerative causes, systemic illness or postsurgical pathology (2). This patient has significant thoracolumbar stenosis. Intraoperatively, we found darkening of the ligamentum flavum with significant melanin deposition, which suggests that the cause of T10/11 segment stenosis is mainly the calcification of the ligamentum flavum (Figure 3). The reason for ossification of the ligamentum flavum may be the deposition of HGA. Additionally, disc degeneration and loss of height may force invagination of the ligamentum flavum and subsequent pressure on the dural dorsal capsule, contributing to hypertrophy of the ligamentum flavum (2).

Currently, there is no effective therapy for AKU, and symptomatic supportive treatment is the mainstay. Dietary restrictions on phenylalanine and tyrosine intake and oral vitamin C are suggested to reduce the production and deposition of HGA (14–16). It has been reported that nitisinone can reduce HGA production and has a therapeutic effect on AKU (17, 18). Surgery is considered feasible for patients with spinal or peripheral large joint involvement to improve the quality of survival (19, 20).

Finally, this case report is a further reminder that orthopedic surgeons must be thorough in their examination and diagnosis and not abandon any suspected diagnoses. Surgery remains an important treatment for this condition.

## Data Availability

The raw data supporting the conclusions of this article will be made available by the authors, without undue reservation.
